# Supplementary material for: Mapping dementia research in Indonesia: A scoping review of evidence, gaps, and future directions
Source: PLOS Glob Public Health. 2026 Mar 9;6(3):e0005444. doi: 10.1371/journal.pgph.0005444 (PMC12970937; doi:10.1371/journal.pgph.0005444)
Supplement: S1 Table — (DOCX) [file pgph.0005444.s002.docx]

S1 Table

| **First Author (Year)** | **Category** | **Diagnosis** | **Type** | **Location** | **Sample size** | **Setting** |
| --- | --- | --- | --- | --- | --- | --- |
| Alzi(2021) | Public Health Priority | DEM | Policy analysis | National | N/A | Non-Governmental Organization |
| Andrews (2024 | Support for Dementia Carers | DEM | Cross-sectional | National | 76 | Community |
| Ardiningrum (2024) | Support for Dementia Carers | DEM | Mixed-Method | Yogyakarta | 65 | Community |
| Asih (2024) | Diagnosis | DEM | Cross-sectional | Bali | 28 | Hospital |
| Astutik (2024) | Risk Reduction | CI | Cross-sectional | East java (Malang) | 155 | Community |
| Azwar (2021) | Risk Reduction | DEM | Case-control | Jakarta | 345 | Outpatient clinic (Hospital) |
| Breuer (2022) | Treatment, Care, and Support | DEM | Qualitative | Multinational (LMIC) | N/A | University |
| Chandra (1992) | Treatment, Care, and Support | DEM | RCT | East Java (Surabaya) | 146 | Hospital & private clinics |
| Chandra (2024) | Risk Reduction | MCI | Cross-sectional | National | 4287 | Population-level |
| Cynthia (2024) | Research and Innovation | MCI | Cross-sectional | Jakarta | 111 | Hospital |
| Dawes (2021) | Awareness and Friendliness | DEM | Cross-sectional | Multinational (Global) | 22 | Nursing home |
| Dewi (2024) | Risk Reduction | CI | Case-control | Bali | 62 | Diabetic centre (Hospital) |
| Erlianti (2023) | Risk Reduction | CI | Cross-sectional | National | 6755 | Population-level |
| Farina (2022) | Treatment, Care, and Support | DEM | Cross-sectional | Jakarta | 17 | Outpatient clinic (Hospital) |
| Farina (2023) | Risk Reduction | DEM | Cross-sectional | Multinational (Global) | 2110 | Community |
| Farina (2024) | Awareness and Friendliness | DEM | Cross-sectional | Multisite (Jakarta/ Medan) | 4413 | Community |
| Fitri (2023) | Risk Reduction | CI | Cross-sectional | Multisite (Jakarta/ Medan) | 211 | Community |
| Fitri (2020) | Risk Reduction | CI | Cross-sectional | Multisite (Jakarta/ Medan) | 38 | Memory Clinic (Hospital) |
| Fitri (2024) | Diagnosis | DEM | Cross-sectional | North Sumatra | 2110 | Population-level |
| Fitri (2024) | Risk Reduction | DEM, CI | Cross-sectional | Multisite (Sumatra) | 1160 | Hospital |
| Fitri (2025) | Diagnosis | DEM | Cross-sectional | North Sumatra (Medan) | 140 | Memory Clinic (Hospital) |
| Fukuhara (2014) | Research and Innovation | FTLD | Case report | Multinational (Asia) | 4 | Outpatient clinic (Hospital) |
| Gajdusek (1982) | Risk Reduction | PD | Cross-sectional | Papua (West New Guinea) | 97 | Community |
| Grenfell-Essam (2018) | Diagnosis | DEM | Cross-sectional | Multisite (Indonesia) | 719 | Community |
| Hadiyoso (2022) | Diagnosis | VD | Case-control | West Java (Bandung) | 50 | Hospital |
| Hadiyoso (2023) | Diagnosis | VD | Cross-sectional | West Java (Bandung) | 84 | Neurological clinic (Hospital) |
| Handajani (2020) | Risk Reduction | MCI | Cross-sectional | Jakarta | 278 | University |
| Handajani (2020) | Treatment, Care, and Support | MCI | Cohort | Jakarta | 90 | Community |
| Handajani (2022) | Risk Reduction | DEM | Cross-sectional | National | 4236 | Community |
| Handajani (2025) | Risk Reduction | CI | Cross-sectional | Jakarta | 334 | Community |
| Harahap (2022) | Risk Reduction | CI | Cross-sectional | West Nusa Tenggara | 155 | Hospitals |
| Harahap (2024) | Risk Reduction | CI | Cross-sectional | West Nusa Tenggara | 114 | Community |
| Heppy (2025) | Risk Reduction | CI | Cross-sectional | West Sumatra | 95 | Community |
| Hestiantoro (2017) | Research and Innovation | MCI | Cross-sectional | Jakarta | 282 | University |
| Hogervorst (2021) | Risk Reduction | DEM | Cohort | Multisite (Indonesia) | 705 | Community |
| Huda (2017) | Diagnosis | AD | Cross-sectional | West Java (Bandung) | 30 | University |
| Iswahyudhi (2023) | Risk Reduction | CI | Cross-sectional | South Sulawesi (Makassar) | 93 | HIV clinic (Hospital) |
| Juanita (2024) | Risk Reduction | DEM | Cross-sectional | North Sumatra (Aceh) | 400 | Community |
| Juniarti (2021) | Treatment, Care, and Support | DEM | RCT | West Java (Bandung) | 90 | Community |
| Kokubo (2022) | Research and Innovation | PD | Case report | Papua (West New Guinea) | N/A | Community |
| Komalasari (2023) | Diagnosis | DEM | Cross-sectional | West Java (Banten) | 135 | Nursing home |
| Kristanti (2018) | Support for Dementia Carers | DEM | Cross-sectional | Yogyakarta | 25 | Outpatient clinic (Hospital) |
| Kristian (2024) | Diagnosis | CI | Cross-sectional | Jakarta | 23 | Community |
| Kurdi (2019) | Treatment, Care, and Support | CI | Cohort | South Sumatra (Palembang) | 60 | Community |
| Lam (2015) | Risk Reduction | AD | Cross-sectional | Multinational (Asia) | 2404 | Community |
| Lam (2021) | Risk Reduction | AD | Cross-sectional | Multinational (Asia) | 162 | Outpatient clinic (Hospital) |
| Langavant (2020) | Risk Reduction | DEM | Cohort | Multinational (Global) | N/A | Research institute |
| Le (2025) | Risk Reduction | DEM | Cross-sectional | National | 6693 | Population-level |
| Lim (2018) | Diagnosis | DEM | Cohort | Multinational (Asia) | 284 | Memory Clinic (Hospital) |
| Maharani (2025) | Treatment, Care, and Support | CI | Quasi-experimental | Jakarta | 22 | Nursing home |
| Maitimoe (2019) | Support for Dementia Carers | AD | Cross-sectional | Multisite (Indonesia) | 50 | Community |
| Maryam (2020) | Risk Reduction | CI | Cross-sectional | Jakarta | 341 | Community |
| Maryam (2021) | Awareness and Friendliness | AD | Cross-sectional | Jakarta | 354 | Community |
| Masengi (2025) | Risk Reduction | CI | Cross-sectional | North Sulawesi (Tomohon) | 97 | Community |
| Miladitiya (2023) | Risk Reduction | MCI | Cross-sectional | East Java (Kediri) | 427 | Community |
| Muhammad (2024) | Risk Reduction | CI | Cross-sectional | Multisite (Central Java) | 409 | Community |
| Mulyani (2022) | Awareness and Friendliness | DEM | Cross-sectional | Yogyakarta | 386 | Hospital |
| Mulyani (2023) | Awareness and Friendliness | DEM | Cross-sectional | Yogyakarta | 103 | Community |
| Mulyani (2025a) | Awareness and Friendliness | DEM | Cross-sectional | Yogyakarta | 115 | Hospital |
| Mulyani (2025b) | Awareness and Friendliness | DEM | Cross-sectional | Yogyakarta | 161 | University |
| Nasrun (2021) | Support for Dementia Carers | DEM | Cross-sectional | Jakarta | 84 | Outpatient clinic (Hospital) |
| Nasrun (2024) | Treatment, Care, and Support | DEM | Cross-sectional | National | 336 | Population-level |
| Nurcaya (2024) | Treatment, Care, and Support | Stroke | Quasi-experimental | South Sulawesi (Makassar) | 45 | Stroke centre (Hospital) |
| Ong (2021) | Risk Reduction | DEM | Cross-sectional | West Java (Sumedang) | 686 | University |
| Pandhita (2020) | Diagnosis | MCI | Cross-sectional | Jakarta | 212 | Community |
| Pradana (2025) | Support for Dementia Carers | DEM | Qualitative | West Java (Bekasi) | 10 | Community |
| Prihandini (2022) | Risk Reduction | DEM | Cross-sectional | East java (Malang) | 84 | Community |
| Putra (2025) | Risk Reduction | CI | Cross-sectional | National | 2313 | Population-level |
| Putri (2021) | Support for Dementia Carers | DEM | Cross-sectional | Multisite (Java) | 250 | Outpatient clinic (Hospital) |
| Putri (2022) | Support for Dementia Carers | DEM | Cross-sectional | Multisite (Java) | 207 | Outpatient clinic (Hospital) |
| Rachmah (2024) | Risk Reduction | DEM | Cross-sectional | Eat Java (Surabaya) | 400 | Community |
| Rahmawati (2024) | Treatment, Care, and Support | DEM | Quasi-experimental | Central Java | 33 | Nursing home |
| Rai (2021) | Treatment, Care, and Support | DEM | Mixed method | Multisite (Jakarta/Depok) | 39 | Community |
| Rohr (2020) | Risk Reduction | AD | Cross-sectional | Jakarta | 260 | University |
| Samatra (2018) | Research and Innovation | mTBI | Cohort | Bali | 70 | A&E, Surgical & Hospital |
| Sari (2021) | Risk Reduction | CI | Cross-sectional | West Java (Bandung) | 35 | Community |
| Sari (2022) | Awareness and Friendliness | DEM | Cross-sectional | National | 6327 | Population-level |
| Sari (2023) | Treatment, Care, and Support | DEM | Quasi-experimental | Central java (Solo) | 60 | University |
| Sari (2024) | Treatment, Care, and Support | DEM | Qualitative | National | 42 | University |
| Sekeon (2020) | Risk Reduction | Stroke | Cross-sectional | North Sulawesi (Manado) | 78 | Neurology ward (Hospital) |
| Semadhi (2023) | Research and Innovation | CI | Cross-sectional | Multisite (Indonesia) | 120 | University |
| Setiyani (2022) | Risk Reduction | CI | Cross-sectional | Central Java (Banyumas) | 99 | Nursing home |
| Shen (2024) | Research and Innovation | AD | Cross-sectional | Jakarta | 128 | University |
| Sitawati (2024) | Support for Dementia Carers | DEM | Cross-sectional | East Java (Surabaya) | 24 | Hospital |
| Situmeang (2016) | Research and Innovation | AD | Cross-sectional | West Java (Banten) | 60 | Memory clinic (Hospital) |
| Sumandar (2024) | Awareness and Friendliness | DEM | Cross-sectional | National | 754 | University |
| Suriastini (2023) | Awareness and Friendliness | DEM | Cross-sectional | Yogyakarta | 121 | Community |
| Susanty (2024) | Risk Reduction | DEM | Cross-sectional | Sulawesi | 200 | Hospital |
| Susianti (2024) | Diagnosis | DEM | Cross-sectional | Yogyakarta | 61 | Memory clinic (Hospital) |
| Suwarni (2018) | Risk Reduction | DEM | Cross-sectional | Central Java (Solo) | 218 | Nursing home |
| Sya'diyah (2022) | Treatment, Care, and Support | DEM | Cross-sectional | East Java (Surabaya) | 40 | Community |
| Sya'diyah (2022) | Treatment, Care, and Support | DEM | Cross-sectional | East Java (Surabaya) | 100 | Community |
| Syafrita (2022) | Research and Innovation | CI | Case-control | West Sumatra (Padang) | 63 | Outpatient clinic (hospital) |
| Tambunan (2023) | Support for Dementia Carers | AD | Qualitative | West Java (Bandung) | 13 | Community |
| Thajeb (2007) | Diagnosis | VD | Case-control | Multinational (Asia) | 161 | University |
| Theresia (2023) | Support for Dementia Carers | DEM | Qualitative | Jakarta | 19 | Community |
| Turana (2014) | Diagnosis | MCI | Cross-sectional | Jakarta | 109 | University |
| Turana (2015) | Research and Innovation | CI | Cross-sectional | Jakarta | 105 | University |
| Turana (2023) | Treatment, Care, and Support | DEM | Cross-sectional | Jakarta | 20 | University |
| Turana (2024a) | Diagnosis | DEM | Cross-sectional | Multinational (LMIC) | 2110 | Community |
| Turana (2024b) | Diagnosis | DEM | Cross-sectional | Multisite (Jakarta/Medan) | 2098 | Community |
| Turana (2024c) | Treatment, Care, and Support | MCI | Quasi-experimental | Jakarta | 22 | Community |
| Utami (2024) | Support for Dementia Carers | CI | Cross-sectional | Yogyakarta | 47 | Memory Clinic (Hospital) |
| Virdyanti (2020) | Risk Reduction | CI | Cross-sectional | Yogyakarta | 143 | Community |
| Widodo (2025) | Research and Innovation | CI | Cross-sectional | Bali | 65 | Hospital |

* AD: Alzheimer’s Disease; CI: Cognitive impairment; DEM: Dementia; FTLD: Frontotemporal lobar degeneration; MCI: Mild cognitive impairment; mTBI: Mild Traumatic Brain Injury; Parkinsonism-related dementias : PD; VD: Vascular dementia; RCT: Randomised control trial.
