# Supplementary material for: Mapping dementia research in Indonesia: A scoping review of evidence, gaps, and future directions
Source: PLOS Glob Public Health. 2026 Mar 9;6(3):e0005444. doi: 10.1371/journal.pgph.0005444 (PMC12970937; doi:10.1371/journal.pgph.0005444)
Supplement: Attachment — Submitted filename: PGPH-D-25-03260 reviewer comment.docx [file pgph.0005444.s003.docx]

**PLOS: reviewers comment for the article number PGPH-D-25-03260**

The scoping review titled “Mapping Dementia Research in Indonesia: A Scoping Review of Evidence, Gaps, and Future Directions” is relevant and timely topic given the period for “WHO Global Action Plan on Dementia (2017–2025)” is just being completed.

The methodological approach is generally sound, and it fits for scoping review. The article describes the details of the methodological processes, and the authors have followed a well-documented standard for scoping review article; and it appears to be transparent and reproducible.

However, given the authors intension to evaluate the existing evidences in line with “WHO Global Action Plan on Dementia (2017–2025)”; the search should probably focus for the period 2015-2025; or should the authors be interested to compare the evidences before and after WHO Global Action Plan, the interval between 2010-2015 and 2016 to 2015 would be more appropriate and informative. Doing so would enable the authors to objectively evaluate the achievements made so far against the “National strategy management of Alzheimer’s and 587 other dementia diseases: towards healthy and productive older persons” and WHO Global action plan 2017 -2025. In addition, the depth of information that can be potentially gained from earlier studies appears to be minimal given there were only a handful of publications for the period 1982-2010.

The organization of the themes, and the way the authors have presented their findings generally matches the suggested thematic organization by the WHO Global Action Plan. However, there were major short comings in the following two areas. A. Missing/ unaccounted References: references 62 -75; and 89-91 were noted cited anywhere in the article. B. There were evidences for contradictory citation in the article, for example, reference number “44’s” tile does not match with frailty while reference “64” may be more fitting for this citation; and reference 80 has been cited for “innovative olfactory screening and FGD-PET imaging correlation.” There is fragmentation of idea when putting the references by category for the information provided under Theme 4 and 5 which has made conceptualization and understanding difficult.

The overall suggestion is to have major revision of the article.
