# Supplementary material for: Mapping dementia research in Indonesia: A scoping review of evidence, gaps, and future directions
Source: PLOS Glob Public Health. 2026 Mar 9;6(3):e0005444. doi: 10.1371/journal.pgph.0005444 (PMC12970937; doi:10.1371/journal.pgph.0005444)
Supplement: Attachment — Submitted filename: Plos Reviewer comment and answer.docx [file pgph.0005444.s005.docx]

Dear Reviewers,

We sincerely thank you for your careful reading of our manuscript and for your constructive and insightful comments. We appreciate your recognition of the relevance and timeliness of the review in relation to the completion of the WHO Global Action Plan on the Public Health Response to Dementia (2017–2025), as well as your positive assessment of the methodological rigor and transparency of the scoping review.

In response to your comment regarding the appropriateness of the search period, we have revised the manuscript to explicitly examine the temporal distribution of included studies in relation to Indonesia’s National Dementia Plan (2016) and the WHO Global Action Plan on the Public Health Response to Dementia (2017–2025). While the search strategy was intentionally not restricted by publication year in keeping with scoping review methodology, we now demonstrate that this decision does not dilute policy relevance. As shown in the revised Table 2, only 7 of the 105 included studies (6.7%) were published before 2016, whereas the vast majority were published during the period aligned with national and WHO dementia strategies.

We have therefore restructured Table 2 using policy-relevant publication periods (1982–2000; 2001–2015; 2016–2020; 2021–2025) and revised the Results and Discussion to highlight that dementia research in Indonesia expanded substantially after 2016. We further interpret this post-2016 expansion in relation to the WHO Global Action Plan domains, noting that despite increased research output, evidence remains heavily concentrated in cross-sectional risk factor studies, with limited evaluation of policy implementation, health system integration, or population-level impact. These revisions allow for an objective assessment of the extent to which the emerging evidence base aligns with national and global dementia policy priorities, while preserving the comprehensive mapping purpose of a scoping review. We believe this strengthens the policy relevance and interpretability of the review, as suggested by the reviewer.

We thank the reviewer for identifying issues related to missing, inconsistent, and contradictory references. We carefully reviewed all citations in Themes 4 and 5 to address concerns regarding incorrect referencing and conceptual fragmentation. Citation mismatches were corrected by ensuring that each reference supports a single, clearly defined claim; in particular, claims regarding advanced imaging were revised to reflect the actual scope of the cited studies, and unsupported statements were removed or rephrased. In addition, all previously unaccounted references (62–75 and 89–91) were cross-checked and are now appropriately cited within the manuscript, or removed where no longer relevant. We also restructured Themes 4 and 5 to move away from categorical listing of studies toward an integrated, concept-driven synthesis. Diagnostic tools are now discussed in relation to their clinical purpose, feasibility, and methodological limitations, with a clearer distinction between scalable screening approaches and more resource-intensive diagnostic methods.

We believe these revisions substantially strengthen the manuscript’s clarity, accuracy, and policy relevance. We are grateful for your detailed feedback, which has significantly improved the quality of the review.

On behalf of the authors,

Anna Tjin
